# Oncological Resectability Criteria for Intrahepatic Cholangiocarcinoma: A Preoperative Framework for Multidisciplinary Management

**DOI:** 10.1245/s10434-025-17776-x

**Published:** 2025-07-09

**Authors:** Jun Kawashima, Miho Akabane, Selamawit Woldesenbet, Diamantis I. Tsilimigras, Yutaka Endo, Kota Sahara, François Cauchy, Federico Aucejo, Hugo P. Marques, Rita Lopes, Andreia Rodriguea, Tom Hugh, Feng Shen, Shishir K. Maithel, Bas Groot Koerkamp, Irinel Popescu, Minoru Kitago, Matthew J. Weiss, Guillaume Martel, Carlo Pulitano, Luca Aldrighetti, George Poultsides, Andrea Ruzzente, Todd W. Bauer, Ana Gleisner, Itaru Endo, Timothy M. Pawlik

**Affiliations:** 1 Department of Surgery, The Ohio State University Wexner Medical Center and James Comprehensive Cancer Center, Columbus, OH USA; 2 Department of Gastroenterological Surgery, Yokohama City University, Yokohama, Japan; 3 Department of Transplant Surgery, University of Rochester Medical Center, Rochester, NY USA; 4 Department of HPB Surgery and Liver Transplantation, Beaujon Hospital, Clichy, France; 5 Department of Hepato-Pancreato-Biliary & Liver Transplant Surgery, Digestive Diseases and Surgery Institute, Cleveland Clinic Foundation, Cleveland, OH USA; 6 Department of Surgery, Curry Cabral Hospital, Lisbon, Portugal; 7 Department of Surgery, The University of Sydney, Sydney, NSW Australia; 8 Department of Surgery, Eastern Hepatobiliary Surgery Hospital, Shanghai, China; 9 Division of Surgical Oncology, Winship Cancer Institution, Emory University, Atlanta, GA USA; 10 Department of Surgery, Erasmus University Medical Centre, Rotterdam, The Netherlands; 11 Department of Surgery, Fundeni Clinical Institute, Bucharest, Romania; 12 Department of Surgery, Keio University, Tokyo, Japan; 13 Department of Surgery, Cancer Institute, Northwell Health, New Hyde Park, NY USA; 14 Department of Surgery, University of Ottawa, Ottawa, ON Canada; 15 Department of Surgery, Royal Prince Alfred Hospital, Camperdown, NSW Australia; 16 Department of Surgery, San Raffaele Hospital, Milan, Italy; 17 Department of Surgery, Stanford University, Stanford, CA USA; 18 Division of General and Hepatobiliary Surgery, University of Verona, Verona, Italy; 19 Department of Surgery, University of Virginia, Charlottesville, VA USA; 20 Department of Surgery, University of Colorado Denver, Denver, CO USA

## Abstract

**Introduction:**

Recent advancements in systemic chemotherapy have fueled debates regarding the feasibility of combining systemic therapy with surgery for advanced intrahepatic cholangiocarcinoma (ICC). However, the absence of consensus on oncological resectability criteria has hindered discussions on optimal multidisciplinary management. This study sought to propose preoperative oncological resectability criteria for ICC.

**Methods:**

Patients undergoing upfront curative-intent hepatectomy for ICC were identified from an international multi-institutional database. Independent tumor-related prognostic factors for overall survival were identified by using multivariable Cox regression and utilized to develop resectability criteria.

**Results:**

Among 953 patients, four independent tumor-related predictors of poor prognosis were identified: lymph node metastasis (LNM) on imaging (HR 1.3, 95% confidence interval [CI] 1.07–1.59), tumor size > 5 cm (hazard ratio [HR] 1.52, 95% CI 1.25–1.85), multinodular lesions (HR 2.03, 95% CI 1.64–2.52), and major vascular invasion (HR 1.64, 95% CI 1.34–2.01). High-risk points were identified based on a point system associated with the hazards of each factor: 1 point each for LNM, tumor size > 5 cm, and major vascular invasion, and 2 points for multinodular lesions. Patients were categorized as resectable (R) for scores of 0–1 or borderline resectable (BR) for scores ≥ 2. Patients with BR disease (n = 385, 40.4%) had markedly worse median overall survival versus individuals with R disease (n = 568, 59.6%) (24.6 months vs. 69.7 months, *p *< 0.001). Validation in an external cohort confirmed these findings.

**Conclusions:**

The proposed preoperatively assessable resectability criteria can help differentiate BR versus R disease among ICC patients. These criteria offer a practical framework for preoperative risk stratification, aiding in treatment planning.

**Supplementary Information:**

The online version contains supplementary material available at 10.1245/s10434-025-17776-x.

Intrahepatic cholangiocarcinoma (ICC) is the second most common type of primary liver cancer accounting for approximately 10–20% of all cases.^1^ Over the past several decades, the global incidence of ICC has risen significantly, posing a growing public health challenge.^2,3^ Surgical resection is currently the only potentially curative-intent treatment; however, long-term outcomes remain suboptimal.^4^ Despite curative-intent resection, overall survival (OS) at 5 years ranges between 25 and 40%.^4^ Moreover, recurrence rates are notably high with 50–80% of patients experiencing recurrence within 2 years, and approximately 25% of patients suffering recurrence within 6 months.^5,6^ These findings underscore the urgent need for strategies that go beyond surgery alone to improve outcomes for ICC patients.^7^

Recent advances in systemic therapies—including cytotoxic chemotherapy, targeted therapy, and immunotherapy—have highlighted the potential of multidisciplinary treatment approaches for ICC.^8^ Given the aggressive nature of ICC, integrating perioperative systemic therapies with surgical resection may have the potential to improve outcomes.^6^ Retrospective propensity score-matched studies have reported survival benefits of neoadjuvant therapy in technically resectable ICC.^9–11^ However, robust criteria to determine when upfront surgery is appropriate versus when systemic therapies should be prioritized remain lacking. The lack of standardized guidelines highlights a critical gap in optimizing treatment strategies for ICC patients.

In pancreatic ductal adenocarcinoma (PDAC), well-established resectability criteria—categorizing patients as resectable (R), borderline resectable (BR), or unresectable (UR)—play a crucial role in guiding therapeutic strategies.^12^ These criteria, based on the degree of local invasion, help identify patients suitable for surgery or neoadjuvant therapies, and are associated with differences in oncological outcomes.^12^ Similarly, resectability criteria for hepatocellular carcinoma (HCC), incorporating morphological factors, such as tumor size, tumor number, major vascular invasion, and extrahepatic spread, provide a preoperative framework for multidisciplinary management.^13,14^ These classifications are simple, objective, and can be determined on preoperative imaging.^12,13^ Given the parallels with other hepatopancreatobiliary malignancies, establishing resectability criteria for ICC may offer an important tool to guide multidisciplinary management strategies. Therefore, the objective of the current study was to develop simple, clinically applicable resectability criteria for ICC, incorporating tumor-related factors that can be assessed through preoperative imaging. By leveraging a large, multi-institutional, international database, we sought to develop a framework to optimize multidisciplinary treatment strategies and improve outcomes for ICC patients.

## Methods

### Data Source and Patient Selection

Patients who underwent curative-intent liver resection for ICC between 2000 and 2023 were identified from the International Intrahepatic Cholangiocarcinoma Study Group database.^6^ Patients were excluded if they had 1) received preoperative systemic chemotherapy; 2) macroscopically positive surgical margins (R2 resection); 3) extrahepatic metastasis, defined as nonregional lymph node (LN) metastases or other distant metastases, based on the American Joint Committee on Cancer (AJCC) 8th edition^15^; 4) missing data on key clinicodemographic characteristics; or 5) no follow-up data. The study was approved by the institutional review boards of all participating institutions.

### Variables and Outcomes

Patient demographic and clinicopathologic variables included age, sex, year of surgery (i.e., 2000–2010, 2011–2023), American Society of Anesthesiologist (ASA) classification, cirrhosis, lymph node status on imaging (i.e., N0: negative, N1: metastatic or suspicious), tumor size, tumor number, portal vein invasion (i.e., Vp0–1, Vp2–4),^13^ hepatic vein invasion (i.e., Vv0–1, Vv2–3),^13^ bile duct invasion (i.e., B0–2, B3–4),^16^ T-category based on AJCC 8th edition,^15^ pathological nodal disease (i.e., N0: negative, N1: positive, Nx: not examined), TNM stage on AJCC 8th edition,^15^ microvascular invasion (MVI), morphological subtype (i.e., MF, mass-forming; IG, intraductal growth; PI, periductal infiltrating; MF+PI, periductal infiltrating plus mass-forming), tumor grade (i.e., well, moderate, poorly, undifferentiated), perineural invasion (PNI), type of surgery (i.e., minor hepatectomy, major hepatectomy), lymphadenectomy, surgical margin, postoperative severe complication, and receipt of adjuvant chemotherapy.

Preoperative LN status was assessed based on imaging studies, including computed tomography (CT), magnetic resonance imaging (MRI), or positron emission tomography (PET)/CT. Lymph nodes were categorized as metastatic based on the following criteria: 1) minimal diameter ≥ 10 mm; 2)minimal diameter < 10 mm but located near the tumor with a contrast pattern similar to the tumor; 3) evidence of extranodal invasion (e.g., fluffing); or 4) positive uptake on PET/CT.^17^ Based on AJCC 8th edition, multifocal ICC included both satellite lesions, defined as additional tumors within the same Couinaud liver segment, and intrahepatic metastases, defined as tumors located across different Couinaud segments or involving both hepatic lobes.^15^ The degree of vascular invasion was described according to the Japanese staging system.^13,16^ Portal vein invasion was categorized as invasion of (or tumor thrombus in) main trunk/contralateral branch (Vp4), first-order branch (Vp3), second-order branch (Vp2), and third-order branch or microscopic invasion (Vp1).^13^ Hepatic vein invasion was categorized as invasion of (or tumor thrombus in) an inferior vena cava (Vv3), a major hepatic vein (Vv2), or a peripheral hepatic vein including microvascular invasion (Vv1).^13^ Biliary invasion was categorized as invasion of (or tumor thrombus in) common bile duct/contralateral branch (B4), first-order biliary tree (B3), second-order biliary tree (B2), and third-order biliary tree or microscopic invasion (B1).^13^ Based on several of the latest guidelines, which describe the association between vascular invasion and both surgical complexity and clinical outcomes, major vascular invasion included Vp2–4 (portal vein invasion of [or tumor thrombus in] first/second-order branches or main trunk/contralateral branch), Vv2–3 (hepatic vein invasion of [or tumor thrombus in] major hepatic veins or the inferior vena cava), or B3–4 (biliary invasion of [or tumor thrombus in] first-order biliary tree or the common bile duct or contralateral branches).^4,16^ Hepatectomy was classified as major (≥ 3 segments) or minor (≤ 2 segments).^18^ Severity of postoperative complications was defined according to the Clavien-Dindo classification system (grade I-V); severe complications were defined as Clavien-Dindo classification ≥ III.^19^

The primary outcome was OS, defined as the time interval between the date of resection to the date of death from any cause or last follow-up. The secondary outcome was recurrence-free survival (RFS), defined as the time elapsed between the date of liver resection and recurrence, either confirmed on biopsy or using evidence of a suspicious lesion on follow-up imaging.

### Statistical Analysis

Descriptive statistics were presented as median values with interquartile ranges (IQR) for continuous variables and as frequencies with percentages for categorical variables. Continuous variables were compared by using the Mann-Whitney *U* or Kruskal-Wallis tests, as appropriate. Categorical variables were compared with the χ^2^ test or Fisher’s exact test. Multiple imputations with chain equations (MICE) procedures were utilized to handle missing values.^20^ Survival was estimated by using the Kaplan-Meier method and log-rank tests.

Cox regression analysis was utilized to assess the association of various clinicopathologic factors with OS. These factors included patient background and tumor-related characteristics identifiable through preoperative imaging studies. Variables significant (*p *< 0.1) on univariate analysis relative to OS were subsequently included in the multivariable model. For the purpose of developing resectability criteria, tumor-related factors, including LN status on imaging, tumor size, tumor number, and major vascular invasion, that remained significant in the multivariable analysis were assigned risk points based on their respective hazard ratios (HR), reflecting their relative association with OS.^7,21^ Log-rank tests were used to determine the optimal cutoff for resectability criteria based on the minimal *P* value method. Patients were categorized based on high-risk points for comparative analyses: 1) 0 points vs. 1–5 points; 2) 0–1 points vs. 2–5 points; 3) 0–2 points vs. 3–5 points; and 4) 0–3 points vs. 4–5 points. The grouping that yielded the smallest *P* value was selected as the cutoff, dividing patients into "resectable (R)" and "borderline resectable (BR)" groups. In addition, cumulative probabilities of mortality were visualized using smoothed curves to compare risk distributions between AJCC staging and the proposed resectability criteria. The proposed resectability criteria were then applied to an external validation cohort to evaluate the ability to stratify patients based on OS, as evidenced by distinct survival distributions between the resectability groups. The external validation cohort from the Eastern Hepatobiliary Surgery Hospital consisted of 371 patients who underwent upfront curative-intent resection for ICC (Supplementary Table 1). Statistical significance was set at α = 0.05. All analyses were performed using R version 4.4.2 (R Project for Statistical Computing, Vienna, Austria).

## Results

### Patient Demographics

Among 953 patients who met inclusion criteria, 481 (50.5%) patients were male and median age was 64 years (IQR 56–71). Roughly one in four patients (n = 237, 24.9%) were diagnosed with metastatic lymph nodes on imaging. Median tumor size was 6.0 cm (IQR 4.0–8.4), and 159 (16.7%) patients had multinodular lesions. Portal vein invasion (Vp2–4) was observed in 116 (12.2%) patients, hepatic vein invasion (Vv2–3) in 69 (7.2%) patients, and bile duct invasion (B3–4) in 201 (21.1%) patients. On final pathology, roughly one-third of patients (n = 378, 39.7%) had Stage I disease: 421 (44.2%) patients had T1 tumors, and 189 (19.8%) patients had nodal metastasis (N1). On histological examination, 347 (36.4%) patients had MVI with PI/MF+PI subtype, poorly or undifferentiated tumors, and PNI being present among 170 (17.8%), 225 (23.6%), and 258 (27.1%) patients, respectively. Most patients (n = 695, 72.9%) underwent major hepatectomy with lymphadenectomy (n = 598, 62.7%); 198 (20.8%) patients had an R1 resection. Postoperatively, 235 (24.7%) patients experienced a severe complication, and 357 (37.5%) patients received adjuvant chemotherapy (Table 1). Among patients who received adjuvant chemotherapy, intravenous gemcitabine-based regimens were administered in 155 (43.4%) patients, whereas 100 (28.0%) patients received oral single-agent therapy with capecitabine or S-1. Additionally, 43 (12.4%) patients received intravenous 5-FU–based regimens, 12 (3.3%) patients received other regimens, and 47 (13.2%) patients had unknown regimens.

**Table 1:** Clinicopathological characteristics of the analytic cohort

Characteristics	All patients
	n = 953
Age, years, median (IQR)	64 [56, 71]
Sex, male	481 (50.5)
Year of surgery, 2011–2023	571 (59.9)
ASA classification, > 2	543 (57.0)
Cirrhosis	75 (7.9)
Lymph node metastasis on imaging	237 (24.9)
Tumor size (cm), median (IQR)	6.0 [4.0, 8.4]
Multinodular lesions	159 (16.7)
Portal vein invasion, Vp2–4	116 (12.2)
Hepatic vein invasion, Vv2–3	69 (7.2)
Bile duct invasion, B3–4	201 (21.1)
*Pathological T category*	
T1	421 (44.2)
T2	224 (23.5)
T3	218 (22.9)
T4	90 (9.4)
*Pathological N category*	
N0	409 (42.9)
N1	189 (19.8)
Nx	355 (37.3)
*Pathological TNM stage*	
I	378 (39.7)
II	166 (17.4)
IIIA	153 (16.1)
IIIB	256 (26.9)
Microvascular invasion	347 (36.4)
Morphologic type, PI/MF+PI	170 (17.8)
Grade, poor/undifferentiated	225 (23.6)
Perineural invasion	258 (27.1)
Major hepatectomy	695 (72.9)
Lymphadenectomy	598 (62.7)
Surgical margin, R1	198 (20.8)
Severe complication	235 (24.7)
Adjuvant chemotherapy	357 (37.5)

Values are (n%) unless otherwise indicated

ASA, American society of Anesthesiologists; PI/MF+PI, periductal infiltrating/mass forming plus periductal infiltrating

To evaluate the validity of preoperative imaging-based nodal assessment, radiographic LN status were compared with pathological findings (N0/Nx vs. N1). The sensitivity and positive predictive value of imaging-detected nodal metastasis were 0.79 and 0.84, respectively. The specificity and negative predictive value were lower (0.39 and 0.31, respectively), and the overall proportion of correctly classified patients was 0.71.

### Defining Resectability Criteria

On multivariable Cox regression, after adjustment for relevant patient and preoperative tumor characteristics, ASA class > 2 (HR 1.29, 95% CI 1.07–1.56, *p *= 0.008), liver cirrhosis (HR 1.59, 95% CI 1.16–2.19, *p *= 0.004), LN metastasis on imaging (HR 1.28, 95% CI 1.04–1.56, *p *= 0.018), tumor size > 5 cm (HR 1.53, 95% CI 1.26–1.86, *p *< 0.001), multinodular lesions (HR 2.03, 95% CI 1.64–2.52, *p *< 0.001), and major vascular invasion (HR 1.64, 95% CI 1.34–2.01, *p *< 0.001) were each independently associated with worse OS. Tumor characteristics identified as independent prognostic factors were assigned “risk” points based on the respective HRs: LN metastasis on imaging, 1 point; tumor size > 5 cm, 1 point; multinodular lesions, 2 points; and major vascular invasion, 1 point (Table 2).

**Table 2:** Univariable and multivariable COX regression analysis for overall survival

	Univariate analysis		Multivariate analysis		Point
Variables	HR [95% CI]	*p*	HR [95% CI]	*p*	
Age	1.00 [1.00, 1.01]	0.337			
*Sex*					
Female	Ref		Ref		
Male	1.16 [0.97, 1.39]	0.098	1.17 [0.97, 1.40]	0.099	
*ASA classification, > 2*					
Classification 1,2	Ref		Ref		
Classification, > 2	1.25 [1.04, 1.49]	0.018*	1.29 [1.07, 1.56]	0.008*	
*Year of surgery, 2011*–*2023*					
2000–2010	Ref				
2011–2023	0.95 [0.79, 1.14]	0.576			
*Cirrhosis*					
No	Ref		Ref		
Yes	1.43 [1.05, 1.96]	0.023*	1.59 [1.16, 2.19]	0.004*	
*Lymph node status on imaging*					
N0	Ref		Ref		
N1	1.43 [1.18, 1.75]	< 0.001*	1.28 [1.04, 1.56]	0.018*	1
*Tumor size (cm)*					
≤ 5	Ref		Ref		
> 5	1.52 [1.26, 1.83]	< 0.001*	1.53 [1.26, 1.86]	< 0.001*	1
*Tumor number*					
Single lesion	Ref		Ref		
Multinodular lesions	2.17 [1.76, 2.68]	< 0.001*	2.03 [1.64, 2.52]	< 0.001*	2
*Major vascular invasion*					
Vp0–1, Vv0–1, and B0–2	Ref		Ref		
Vp2–4, Vv2–3, or B3–4	1.51 [1.24, 1.83]	< 0.001*	1.64 [1.34, 2.01]	< 0.001*	1

ASA, American society of Anesthesiologists

*p < 0.05

**Fig. 1:**
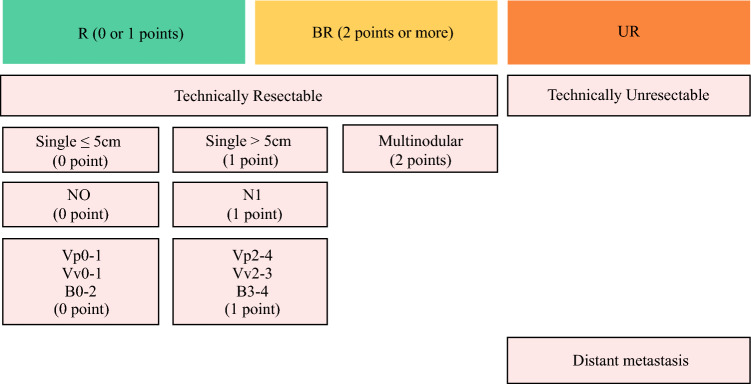
Summary of the proposed resectability criteria. *R* resectable; *BR* borderline resectable; *UR* unresectable

**Fig. 2:**
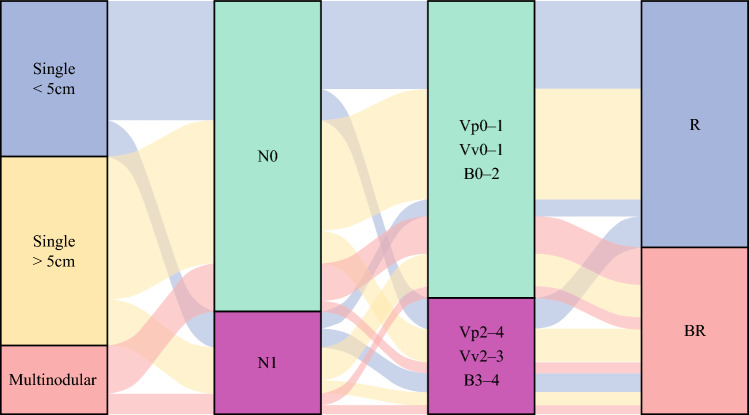
Sankey diagram illustrating the distribution of patients across proposed resectability criteria based on tumor-related factors in the analytic cohort. *R* resectable; *BR* borderline resectable

Cumulative point totals were associated with OS. For example, with a median follow-up of 22.6 months (IQR 9.5–48.8), median OS was 82.3 months (95% CI 67.3–not reached) among patients with 0 points (n = 203, 21.3%), 60.6 months (95% CI 46.2–83) for 1 point (n = 365, 38.3%), 30.3 months (95% CI 26.4–41.7) for 2 points (n = 217, 22.8%), 17.7 months (95% CI 14–26.1) for 3 points (n = 111, 11.6%), and 19.2 months (95% CI 15.2–26.1) for 4 or 5 points (n = 57, 6%) (*p *< 0.001) (Supplementary Fig. 1). After evaluating a range of cutoffs relative to OS, high-risk points of 2 or more was defined as BR (*p* = 7.782 × 10^−19^) (Supplementary Table 2). Based on existing guidelines, patients with technically unresectable or extrahepatic metastases were defined as UR.^4,16^ Figure 1 summarizes the proposed resectability criteria. Patients with multinodular ICC, which corresponded to 2 points, were categorized as BR. Furthermore, single tumors were also defined as BR if two or more of the remaining three factors—LN metastasis on imaging, tumor size > 5 cm, and major vascular invasion—were present. Figure 2 depicts the distribution of patients across resectability criteria based on tumor-related factors in the analytic cohort.

### Association Between the Proposed Resectability Criteria and Survival

In the analytic cohort, 568 (59.6%) patients were categorized as R and 385 (40.4%) patients as BR. Postoperative pathological findings indicated more advanced disease among patients preoperatively classified into the BR cohort. Specifically, T3/T4 tumors were more common (n = 161, 41.8% vs. n = 147, 25.9%, *p* < 0.001), and there was a higher incidence of pathological N1 nodes (n = 100, 26% vs. n = 89, 15.7%, *p* < 0.001) and stage IIIB disease (n = 134, 34.8% vs. n = 122, 21.5%, *p* < 0.001). MVI (n = 159, 41.3% vs. n = 188, 33.1%, *p* = 0.012), PI/MF+PI type (n = 93, 24.2% vs. n = 77, 13.6%, *p* < 0.001), poor/undifferentiated grade (n = 107, 27.8% vs. n = 118, 20.8%, *p* = 0.015), and PNI (n = 120, 31.2% vs. n = 138, 24.3%, *p* = 0.023) were also more common among patients classified preoperatively as BR (Table 3).

**Table 3:** Clinicopathological characteristics comparing patients with resectable and borderline resectable

Characteristics	R	BR	*p*
	n = 568 (59.6%)	n = 385 (40.4%)	
Age, years, median (IQR)	64 [56, 71]	63 [55, 71]	0.259
Sex, male	285 (50.2)	196 (50.9)	0.876
Year of surgery, 2011–2023	340 (59.9)	231 (60)	1.000
ASA classification, > 2	325 (57.2)	218 (56.6)	0.908
Cirrhosis	49 (8.6)	26 (6.8)	0.352
Lymph node metastasis on imaging	39 (6.9)	198 (51.4)	< 0.001*
Tumor size (cm), median (IQR)	5.0 [3.5, 7]	7 [5.4, 10]	< 0.001*
Tumor number, median (IQR)	1 [1]	1 [1, 2]	< 0.001*
Portal vein invasion, Vp2–4	34 (6)	82 (21.3)	< 0.001*
Hepatic vein invasion, Vv2–3	32 (5.6)	37 (9.6)	0.028*
Bile duct invasion, B3–4	51 (9)	150 (39)	< 0.001*
Pathological T category			< 0.001*
T1	320 (56.3)	101 (26.2)	
T2	101 (17.8)	123 (31.9)	
T3	104 (18.3)	114 (29.6)	
T4	43 (7.6)	47 (12.2)	
Pathological N category			< 0.001*
N0	241 (42.4)	168 (43.6)	
N1	89 (15.7)	100 (26)	
Nx	238 (41.9)	117 (30.4)	
Pathological TNM stage			< 0.001*
I	293 (51.6)	85 (22.1)	
II	79 (13.9)	87 (22.6)	
IIIA	74 (13)	79 (20.5)	
IIIB	122 (21.5)	134 (34.8)	
Microvascular invasion	188 (33.1)	159 (41.3)	0.012*
Morphologic type, PI/MF+PI	77 (13.6)	93 (24.2)	< 0.001*
Grade, poor/undifferentiated	118 (20.8)	107 (27.8)	0.015*
Perineural invasion	138 (24.3)	120 (31.2)	0.023*
Major hepatectomy	370 (65.1)	325 (84.4)	< 0.001*
Lymphadenectomy	330 (58.1)	268 (69.6)	< 0.001*
Surgical margin, R1	113 (19.9)	85 (22.1)	0.463
Severe complication	123 (21.7)	112 (29.1)	0.011*

Values are (n%) unless otherwise indicated

R, resectable; BR, borderline resectable; ASA, American society of Anesthesiologists; PI/MF+PI, periductal infiltrating/mass forming plus periductal infiltrating

*p < 0.05

**Fig. 3:**
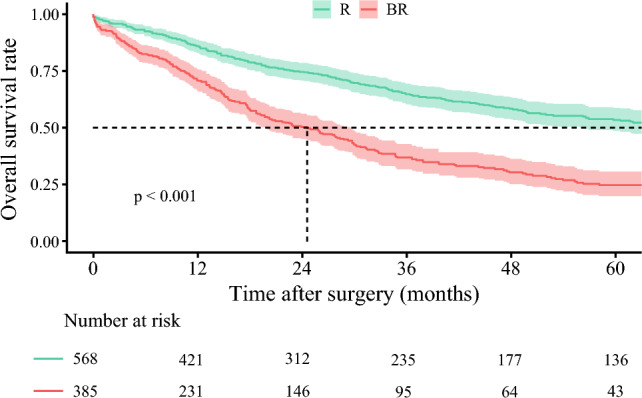
Kaplan-Meier curves comparing overall survival between patients with R and BR in the analytic cohort. *R* resectable; *BR* borderline resectable

**Fig. 4:**
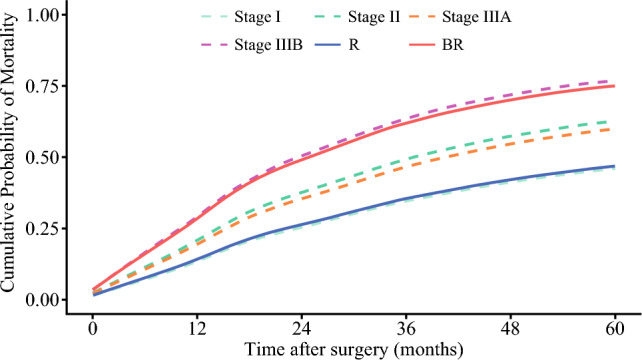
Cumulative probability of mortality stratified by AJCC staging system (Stage I, II, IIIA, IIIB) and the proposed resectability criteria. *AJCC* American Joint Committee on Cancer; *R* resectable; *BR* borderline resectable

Patients classified as BR preoperatively had worse survival outcomes compared with individuals deemed to have R disease. The median OS among patients preoperatively classified as BR was only 24.6 months (95% CI 19.7–29.8), which was markedly shorter than the 69.7 months (95% CI 56.6–93.2) observed among patients with R disease (*p *< 0.001) (Fig. 3). Similarly, the median RFS was shorter among patients with disease preoperatively classified as BR (13 months [95% CI 11.6–17.1]) versus individuals with R category disease (34.3 months [95% CI 27.9–48.3]) (*p* < 0.001) (Supplementary Fig. 2). Figure 4 depicts the cumulative probability of mortality stratified by AJCC staging system (Stage I, II, IIIA, IIIB) relative to the proposed resectability criteria. Of note, individuals with disease classified as BR in the preoperative setting had cumulative mortality that was comparable to pathological Stage IIIB disease, whereas patients with R disease demonstrated survival similar to Stage I disease.

### Validation of Resectability Criteria in the External Cohort

Among 371 patients in the external validation cohort, 305 (82.2%) patients were categorized with R disease and 66 (17.8%) patients had BR disease (Supplementary Table 1). After a median follow-up of 23.6 months (IQR 15.9–37), patients with disease categorized preoperatively as BR versus R had a worse median OS (25.7 months, 95% CI 19.8–35.1 vs. 66.2 months, 95% CI 48.7–not reached, respectively; *p* < 0.001) (Supplementary Fig. 3). A similar trend was noted for RFS; patients with preoperative BR disease had a median RFS of only 7.5 months (95% CI 4.4–15.4) versus 60.6 months (95% CI 26.4–not reached) among individuals who had R disease (*p* < 0.001) (Supplementary Fig. 4).

## Discussion

While curative-intent liver resection followed by adjuvant chemotherapy remains the cornerstone of treatment for patients with ICC, 50–80% of patients experience postoperative disease recurrence.^4–6^ Given the aggressive nature of ICC, high recurrence rates, and the poor prognosis even among patients with favorable conditions undergoing curative-intent resection, there is a need to reconsider the current management approach for resectable disease.^7^ One critical role of preoperative systemic therapy is to identify and stratify patients based on their suitability for surgery.^22^ Therefore, a paradigm shift from upfront surgery to preoperative therapy is reasonable, particularly for patients at high risk of poor prognosis.^7^ To facilitate this shift, concise preoperative criteria that can reliably stratify patients based on expected prognosis are essential. While resectability criteria have been well-established for PDAC and recently proposed for HCC, no such criteria currently exist for ICC.^12–14^ As such, the current study was important because we proposed oncological resectability criteria for ICC based on tumor characteristics, including tumor size, tumor number, LN status, and major vascular invasion—all of which can be assessed based on preoperative imaging. Patients classified with BR disease had a markedly worse survival with upfront resection compared with individuals who had R disease (median OS 24.6 months, 95% CI 19.7–29.8 vs. 69.7 months, 95% CI 56.6–93.2, *p* < 0.001). These data were validated in an external cohort of patients with ICC (Supplementary Fig. 4). In turn, analogous to pancreatic cancer, we proposed novel resectability criteria categories (i.e., R, resectable; BR, borderline resectable; UR, unresectable) to assess patients in the preoperative setting (Fig. 1). By identifying high-risk patients in the preoperative setting using the proposed framework, surgeons can better optimize treatment plans and apply an evidenced-based approach to help decide on upfront resection versus preoperative systemic therapy, which can improve patient outcomes.

The proposed resectability criteria consisted of four tumor-related factors: tumor number, tumor size, LN status, and major vascular invasion—all of which are well-established prognostic factors associated with ICC.^23–33^ Multinodular ICC often reflects early intrahepatic dissemination driven by biological mechanisms such as PNI, portal vein involvement, and the IG pattern.^23^ These processes often occur before LN or distant hematogenous spread, underscoring the aggressive nature of multinodular disease.^3^ Several studies have reported worse outcomes for patients with multinodular ICC, questioning the utility of upfront surgical resection in this subgroup of individuals.^23–26^ Notably, in the modified AJCC 8th edition staging system proposed by the European Network for the Study of Cholangiocarcinoma group, patients with multinodular disease were classified as stage IVa; patients with multinodular disease had survival outcomes markedly worse than individuals with solitary tumors, even those with advanced T3/T4 or N1 disease.^23^ Consistent with these findings, data from the current study demonstrated that multinodular lesions were associated with the highest hazards of mortality among the clinical and pathological factors investigated. Given the strong association on OS, multinodular ICC was defined as BR in the current criteria, reflecting its biological aggressiveness and association with very poor outcomes. In turn, patients with multinodular ICC should be categorized as BR and strongly considered for preoperative systemic therapy.

Tumor size > 5 cm has also been recognized as a key prognostic criterion in the AJCC staging system, which distinguishes T1a (≤ 5 cm) from T1b (> 5 cm).^15,27^ Larger tumor size may be associated with more aggressive tumor biology, including higher risk of MVI and intrahepatic dissemination, both of which can contribute to poor outcomes.^28^ In the present study, patients with tumors > 5 cm had a 53% higher likelihood of mortality compared with individuals with tumors ≤ 5 cm, underscoring the importance of tumor size as a preoperative mortality risk factor. LN metastasis, based on preoperative imaging, was also independently associated with worse OS. Although sometimes challenging to diagnosis owing to limitations in imaging accuracy, preoperative identification of metastatic LN is a powerful predictor of long-term outcomes.^7,16,29^ Indeed, Tsilimigras et al. reported that preoperative LN status was an independent determinant of early recurrence, supporting its inclusion as a key criterion for risk stratification.^7^ In the current study, imaging-based nodal assessment demonstrated relatively low specificity and negative predictive value, suggesting that some patients with occult nodal disease may not be correctly identified preoperatively. Nonetheless, its high sensitivity and positive predictive value indicate good performance in identifying patients with true nodal disease, supporting its clinical utility for risk stratification and treatment decision-making. Major vascular invasion, such as portal vein, hepatic vein, and bile duct invasion, is yet another hallmark of advanced local disease and has been strongly associated with poor survival outcomes.^4,16^ Previous studies have demonstrated that hepatic vein and portal vein involvement are independently associated with OS.^30–33^ Similarly, Bile duct invasion is associated with worse prognosis.^16,33^ For example, Orimo et al. reported that ICC patients with major bile duct invasion had a 5-year OS of 26.8% versus 56.8% among individuals without bile duct involvement.^33^ In turn, the proposed resectability criteria incorporated preoperative tumor number, tumor size, LN status, and major vascular invasion, which provided a comprehensive and practical clinical framework to risk stratify ICC patients relative to resection. While patients with a solitary ICC tumor should be considered as R disease, patients with single tumors that also had two or more of the remaining three factors (i.e., LN metastasis on imaging, tumor size > 5 cm, and major vascular invasion) should be deemed as BR.

Interestingly, mortality of patients classified as BR closely aligned with that of AJCC Stage IIIB disease, while patients with R disease had outcomes similar to Stage I disease.^15^ Importantly, these data strongly suggested that the proposed resectability criteria, which were based on preoperative imaging, correlated strongly with the AJCC staging system that relies on postoperative pathological evaluation.^15^ Of note, patients with BR disease had a median OS of only two years and a median RFS of approximately one year, demonstrating the very poor prognosis in this subset of individuals. These results were further validated in an external cohort, which demonstrated that the criteria were generalizable and reproducible. In turn, the proposed resection criteria can be used to stratify patients for upfront resection or preoperative neoadjuvant chemotherapy, which can treat micrometastasis and delineate tumor biology.^34^ Indeed, several studies have reported improved outcomes among patients with high-risk cholangiocarcinoma treated with preoperative systemic therapy.^9–11^ For instance, Yadav et al. reported that patients who received neoadjuvant chemotherapy followed by surgery achieved a median OS of 40.3 months versus 32.8 months among patients treated with upfront surgery and adjuvant therapy.^9^ Similarly, Utuama et al. demonstrated a survival advantage among ICC patients with stage II and III disease treated with neoadjuvant therapy with an 40% decreased risk of mortality long-term.^10^ In addition, the NEO-GAP study, a phase II single-arm prospective feasibility trial, recently evaluated preoperative gemcitabine, cisplatin, and nab-paclitaxel among patients with high-risk but technically resectable ICC.^7^ The trial achieved its primary endpoint, confirming that this neoadjuvant regimen was both safe and practical before ICC resection without compromising perioperative outcomes.^7^ Furthermore, several other clinical trials are currently underway to assess the efficacy of neoadjuvant chemotherapy for ICC.^35,36^ The results of these randomized controlled trials are eagerly awaited and may provide critical insights into the role of neoadjuvant therapy to optimize outcomes of ICC patients. Beyond systemic chemotherapy, emerging therapies, including molecular targeted therapies and immunotherapy, are shaping the future treatment landscape for ICC with potential application in the neoadjuvant setting.^34,37,38^ The proposed resectability criteria provide a clinically practical tool to guide treatment decisions, particularly in identifying BR patients who may derive the greatest benefit from upfront resection versus preoperative systemic therapy.

Several limitations should be acknowledged when interpreting the findings of the current study. As a retrospective analysis, there may have been residual selection bias. In addition, while the inclusion of multiple centers was a strength, there may have been some variability in treatment strategies across different institutions. Specifically, differences in surgical techniques and the management of patients preoperatively and postoperatively could have contributed to variability in outcomes. Importantly, this study did not compare outcomes between upfront surgery and neoadjuvant therapy within either the R or BR groups. As such, our findings cannot determine whether preoperative systemic therapy should be limited only to BR patients, nor can the data confirm that upfront surgery is appropriate for all patients classified as R. While the study identified BR patients as having markedly worse survival outcomes, whether multidisciplinary treatment strategies such as neoadjuvant chemotherapy are effective in improving survival requires further study.

## Conclusions

The proposed preoperatively assessable resectability criteria, which incorporated tumor size, tumor number, LN status, and major vascular invasion, can help differentiate BR versus R disease among ICC patients. These criteria offer a practical framework for preoperative risk stratification, aiding in treatment planning and identifying candidates for neoadjuvant therapies. Patients classified as BR demonstrated markedly worse survival outcomes versus individuals with R disease, underscoring the importance of tailored, multidisciplinary approaches for high-risk patients. Further prospective studies are necessary to validate these findings and refine treatment strategies, particularly regarding the integration of systemic therapies. The proposed criteria hold promise to optimize treatment plans, improving surgical outcomes, and enhancing long-term survival among ICC patients.

## Supplementary Information

Below is the link to the electronic supplementary material.

Supplementary file1 (DOCX 32 KB)

## Data Availability

Study data are not publicly available as they contain patient-level personal information but are available from the corresponding author on reasonable request.
