# Supplementary material for: Oncological Resectability Criteria for Intrahepatic Cholangiocarcinoma: A Preoperative Framework for Multidisciplinary Management
Source: Ann Surg Oncol. 2025 Jul 9;32(10):7141–51. doi: 10.1245/s10434-025-17776-x (PMC12454504; doi:10.1245/s10434-025-17776-x)

**Supplementary Table 1**. Clinicopathological characteristics of the external validation cohort.

| Characteristics | All patients | R | BR | *P* value |
| --- | --- | --- | --- | --- |
|  | n=371 | n=305 (82.2%) | n=66 (17.8%) |  |
| Age, years, median (IQR) | 55 [47, 63] | 56 [47, 63] | 54 [46, 58] | 0.110 |
| Sex, male | 257 (69.3) | 216 (70.8) | 41 (62.1) | 0.214 |
| Year of surgery, 2011-2023 | 229 (61.7) | 190 (62.3) | 39 (59.1) | 0.729 |
| ASA classification, >2 | 2 (0.5) | 2 (0.7) | 0 (0.0) | 1.000 |
| Cirrhosis | 85 (22.9) | 70 (23.0) | 15 (22.7) | 1.000 |
| Lymph node metastasis on imaging | 45 (12.1) | 13 (4.3) | 32 (48.5) | **<0.001** |
| Tumor size (cm), median (IQR) | 5.5 [3.8, 7.4] | 5.0 [3.5, 7.1] | 6.8 [5.6, 9.4] | **<0.001** |
| Tumor number, median (IQR) | 1 [1, 1] | 1 [1, 1] | 1 [1, 2] | **<0.001** |
| Portal vein invasion, Vp2-4 | 19 (5.1) | 3 (1.0) | 16 (24.2) | **<0.001** |
| Hepatic vein invasion, Vv2-3 | 4 (1.1) | 2 (0.7) | 2 (3.0) | 0.300 |
| Bile duct invasion, B3-4 | 4 (1.1) | 2 (0.7) | 2 (3.0) | 0.300 |
| Pathological T category |  |  |  | **<0.001** |
| T1 | 294 (79.2) | 269 (88.2) | 25 (37.9) |  |
| T2 | 6 (1.6) | 6 (2.0) | 0 (0.0) |  |
| T3 | 44 (11.9) | 21 (6.9) | 23 (34.8) |  |
| T4 | 27 (7.3) | 9 (3.0) | 18 (27.3) |  |
| Pathological N category |  |  |  | **<0.001** |
| N0 | 54 (14.6) | 44 (14.4) | 10 (15.2) |  |
| N1 | 37 (10.0) | 12 (3.9) | 25 (37.9) |  |
| Nx | 280 (75.5) | 249 (81.6) | 31 (47.0) |  |
| Pathological TNM stage |  |  |  | **<0.001** |
| I | 272 (73.3) | 261 (85.6) | 11 (16.7) |  |
| II | 6 (1.6) | 6 (2.0) | 0 (0.0) |  |
| IIIA | 36 (9.7) | 18 (5.9) | 18 (27.3) |  |
| IIIB | 57 (15.4) | 20 (6.6) | 37 (56.1) |  |
| Microvascular invasion | 38 (10.2) | 30 (9.8) | 8 (12.1) | 0.740 |
| Morphologic type, PI/MF+PI | 1 (0.3) | 1 (0.3) | 0 (0.0) | 1.000 |
| Grade, poor/undifferentiated | 10 (2.7) | 9 (3.0) | 1 (1.5) | 0.815 |
| Perineural invasion | 10 (2.7) | 7 (2.3) | 3 (4.5) | 0.546 |
| Major hepatectomy | 75 (20.2) | 60 (19.7) | 15 (22.7) | 0.696 |
| Lymphadenectomy | 91 (24.5) | 56 (18.4) | 35 (53.0) | **<0.001** |
| Surgical margin, R1 | 54 (14.6) | 41 (13.4) | 13 (19.7) | 0.265 |

Values are (n%) unless otherwise indicated.

Abbreviations: **R**, resectable; **BR**, borderline resectable; **ASA,** American society of Anesthesiologists; **PI/MF+PI,** periductal infiltrating/ mass forming plus periductal infiltrating; Bold font signify *P* value <0.05

**Supplementary Table 2**. Evaluated cutoffs for defining R and BR

| Evaluated Cutoff | *P* value | R | |  | BR | |
| --- | --- | --- | --- | --- | --- | --- |
|  |  | n | OS (mo) |  | n | OS (mo) |
| 0 points vs. 1-5 points | 1.176 × 10^-6^ | 203 | 82.3 |  | 705 | 34.7 |
| **0-1 points vs. 2-5 points** | **7.782 × 10^-19^** | **568** | **69.7** |  | **385** | **24.6** |
| 0-2 points vs. 3-5 points | 2.220 × 10^-16^ | 785 | 51.4 |  | 168 | 17.9 |
| 0-3 points vs. 4-5 points | 8.094 × 10^-8^ | 896 | 47.2 |  | 57 | 19.2 |

Abbreviations: **R**, resectable; **BR**, borderline resectable; **OS**, overall survival

**Supplementary Fig. 1** Kaplan-Meier curves of overall survival according to high-risk points in the analytic cohort


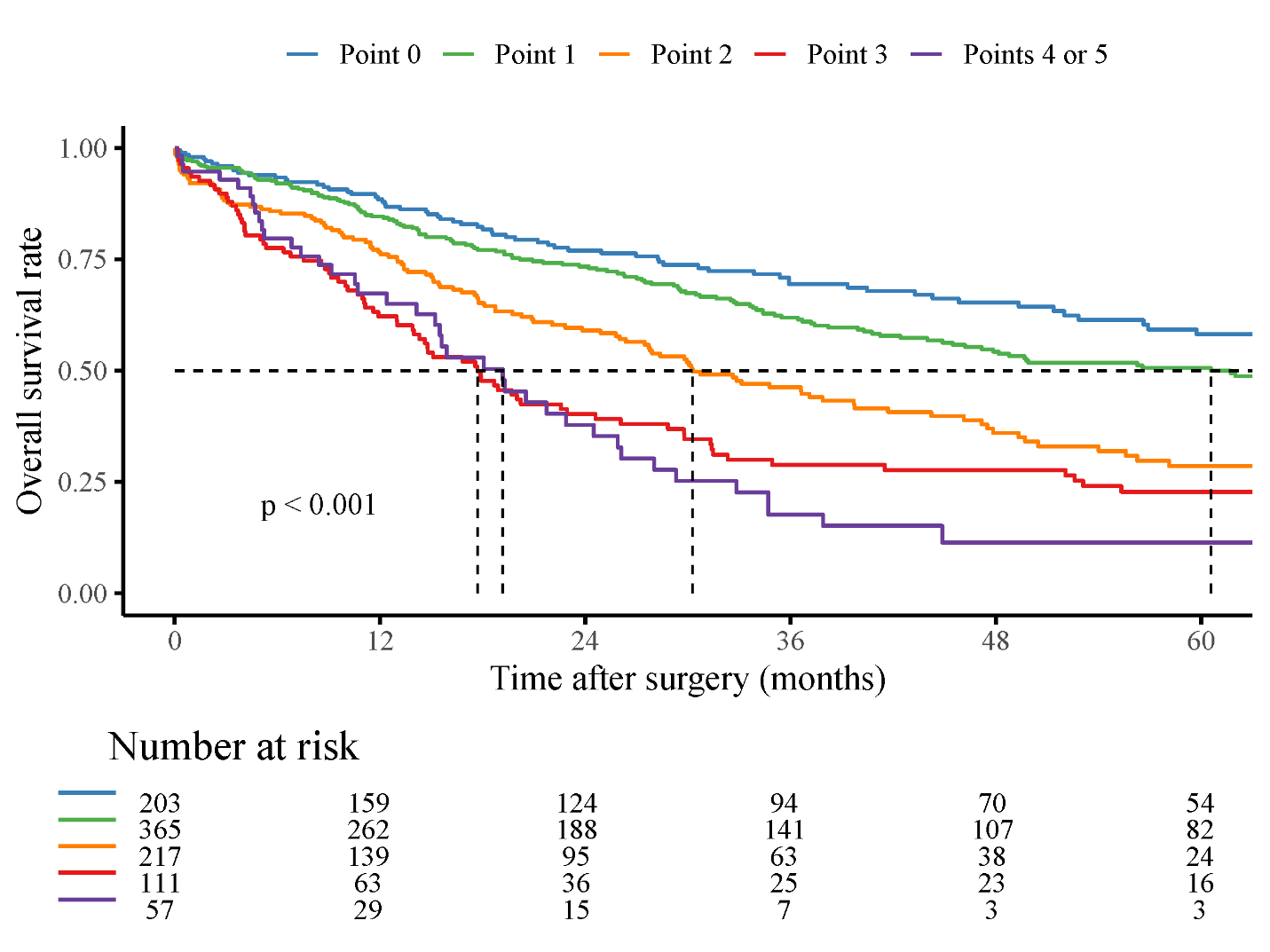


**Supplementary Fig. 2** Kaplan-Meier curves comparing recurrence-free survival between patients with R and BR in the analytic cohort. *R* resectable; *BR* borderline resectable


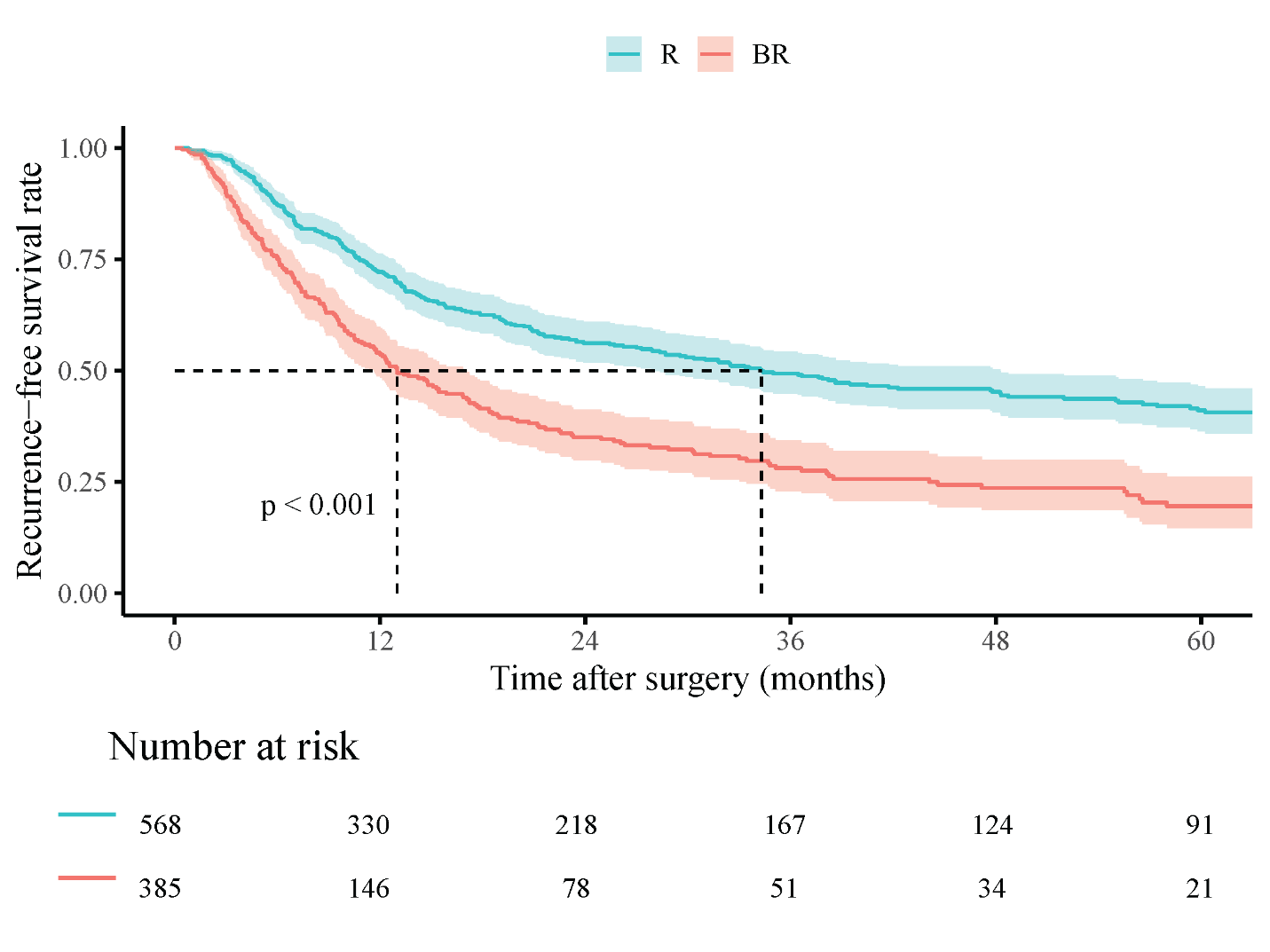


**Supplementary Fig. 3** Kaplan-Meier curves comparing overall survival between patients with R and BR in the external validation cohort. *R* resectable; *BR* borderline resectable


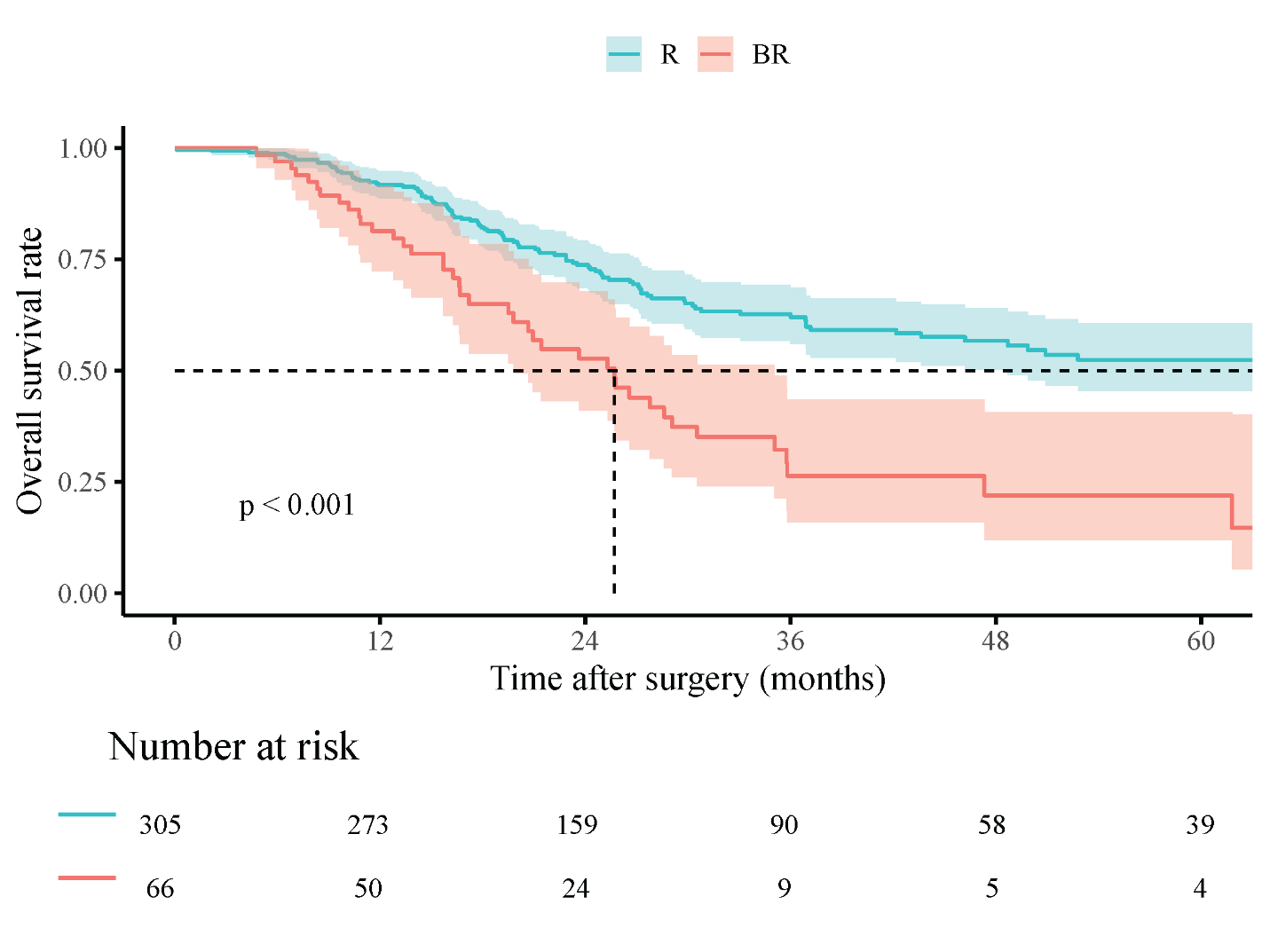


**Supplementary Fig. 4** Kaplan-Meier curves comparing recurrence-free survival between patients with R and BR in the external validation cohort. *R* resectable; *BR* borderline resectable


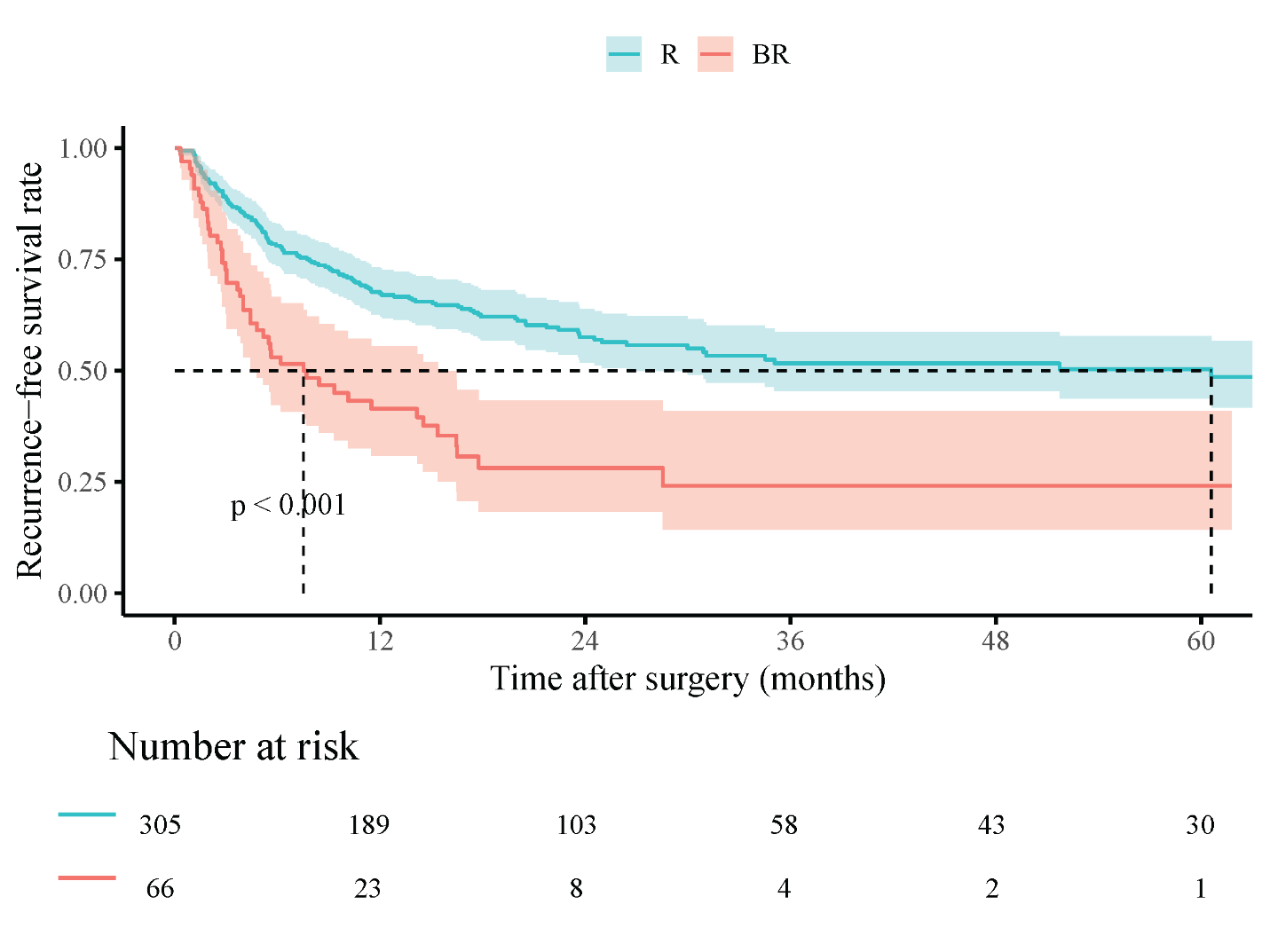

Supplement: Supplementary file 1 — Supplementary file1 (DOCX 32 KB) [file 10434_2025_17776_MOESM1_ESM.docx]
